# Supplementary material for: Portable Iontophoresis Device for Efficient Drug Delivery
Source: Bioengineering (Basel). 2023 Jan 9;10(1):88. doi: 10.3390/bioengineering10010088 (PMC9854461; doi:10.3390/bioengineering10010088)
Supplement: Supplementary file 1 [file bioengineering-10-00088-s001.zip › bioengineering-2063980-Supplementary.pdf]

Supporting information

The experiment shown in Figure S1 was performed to confirm the iontophoresis effect and the direction of the positive and negative charges of the galvanic ionic device. If the current flowed, the permeation of the rhodamine B solution, applied circularly to the FCM epidermis, was expected to be observed. Figure S1(a) is the experimental environment image, and Figure S1(b) is an enlarged image after 20 min. Figure S1(c-d) shows the cross-sectional images of the porcine cadaver skin tissue. In the above method, the permeation for the current effect could not be confirmed. Therefore, the experiment was modified, as shown in Figure S2

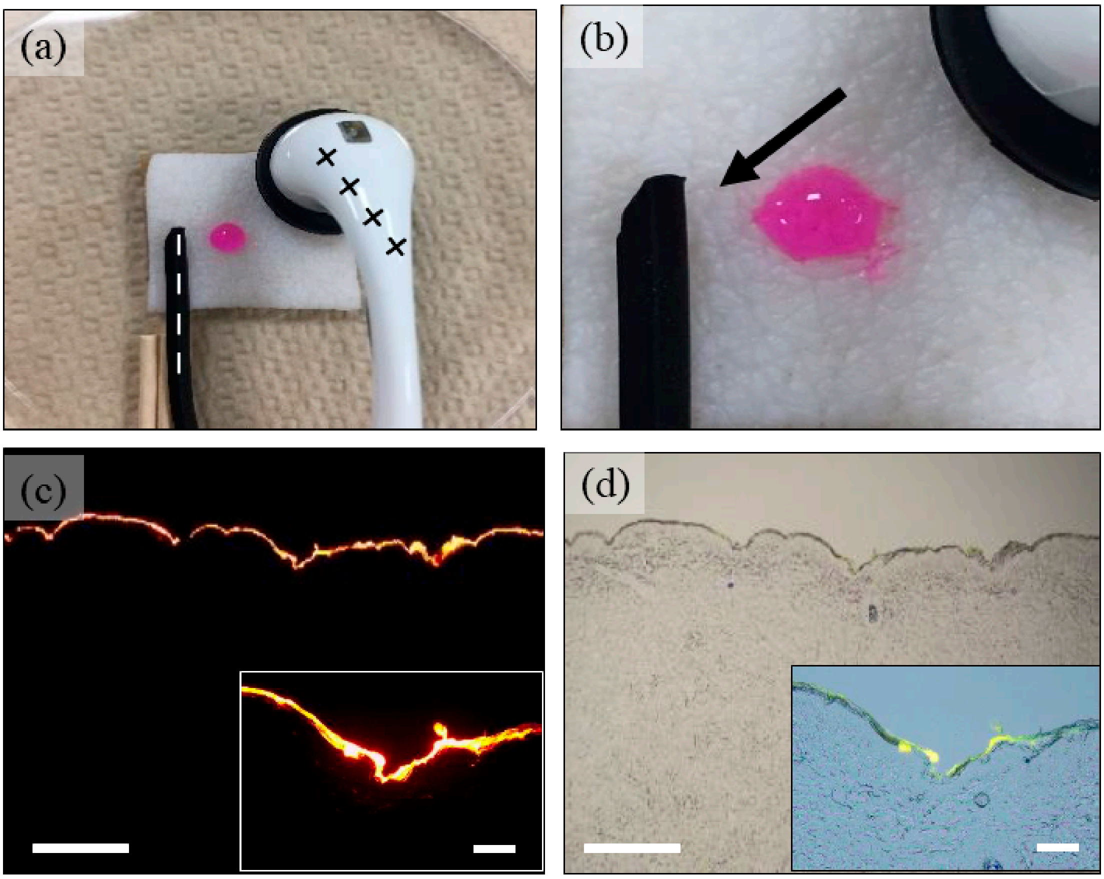

Figure S1. Experiment to determine the + /- charge direction of a galvanic ion device. (a) The experimental image. (b) An enlarged image after 20 min; the arrow points in the direction of the electric field. (c) Fluorescent image of the skin cross-sectional tissue. (d) Fluorescent and optical images of the skin tissue merged. (Scale bars: 500 µm, 100 µm).

The rhodamine B solution was placed as close as possible to the galvanic ionic device for the smooth current flow. The device was placed in the vertical structure to form the electric field distribution in the vertical direction. Figure S2(b) is an enlarged image after 20 min, no iontophoresis phenomenon was observed in this method, see Figure S2(c–d).

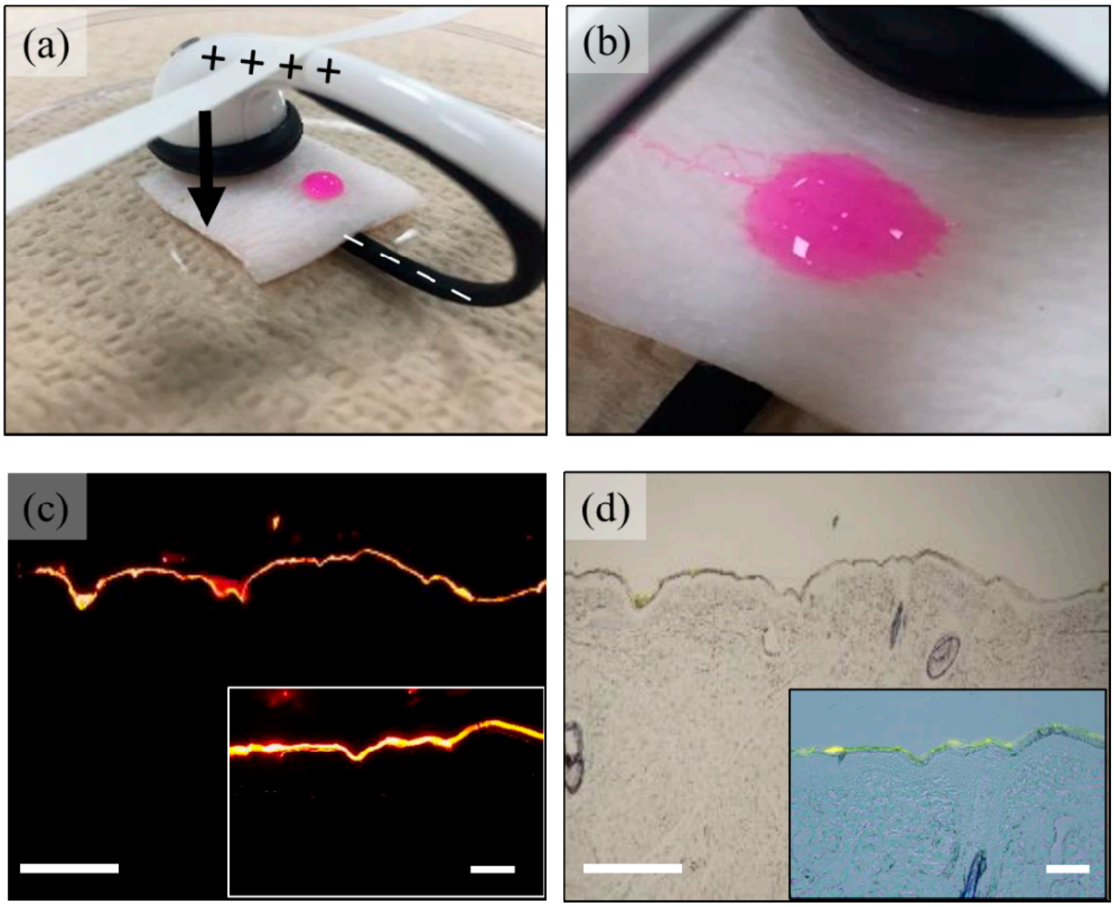

Figure S2. Experiment with galvanic ion devices set, in the vertical direction, as close as possible to the rhodamine B solution. (a) The experimental image. (b) An enlarged image after 20 min; the arrow points in the direction of the electric field. (c) Fluorescent image of the skin cross-sectional tissue. (d) Fluorescent and optical images of the skin tissue merged. (Scale bars: 500 μm, 100 μm) .

Therefore, the experiment was modified by putting the rhodamine B solution in direct contact with the galvanic ionic device to maximize the current flow, as shown in figure S3. Figure S3(a) shows an image of the experimental environment. The effect of iontophoresis was observed (Figure S3(b-c)). We used foil that acted as an electrode between the FCM and the ionic device for the smooth current flow because only a few of the direct contacts with the rhodamine solution were permeable. Figure S3(d–e) shows the experimental environment image, and Figure S3(f) shows the structure and equivalent circuit according to the test conditions. By using the electrode, the current flow was smoothed by lowering the contact resistance between the ionic device and the rhodamine solution, see Figure S3(f). Figure S3(g-l) confirmed the diffusion of rhodamine B into the skin tissue. Therefore, the results confirmed that the current flow was improved by using an electrode between the solution and the ionic device.

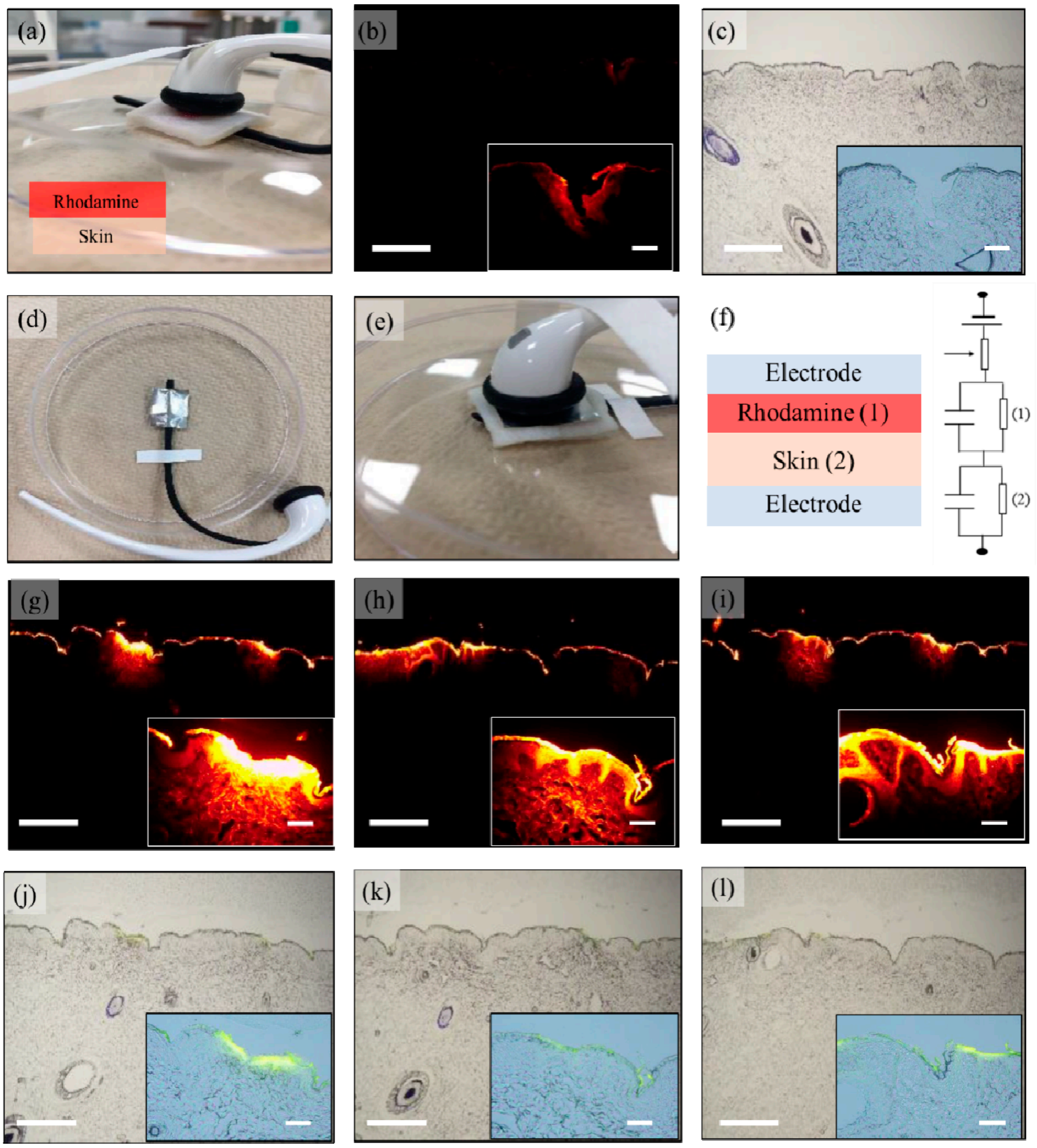

Figure S3. Rhodamine B solution contact experiment between the FCM and the ionic device for smooth current flow. (a) Real experimental images of direct rhodamine contact on the skin without electrodes. (b) Fluorescence images without electrodes. (c) Fluorescence and optical merged images without electrodes. (d,e) Electrode preparation experimental and loading environment images for smooth current flow. (f) Schematic diagram of the experimental condition and equivalent circuit model of resistance and capacitance; the arrow indicates contact resistance. (g-l) Images of fluorescence and optical merged when the electrode is placed. (Scale bars: 500 μm, 100 μm).

In order to observe the application effect of the electric mask pack, an experiment was conducted by composing a device with electrode/FCM/Rhodamine B/electric mask pack. In the case of microscopic fluorescence imaging, the fluorescence expression concentration was significantly lower than the previous experimental results. That is, the essence of the electric mask pack attached to the

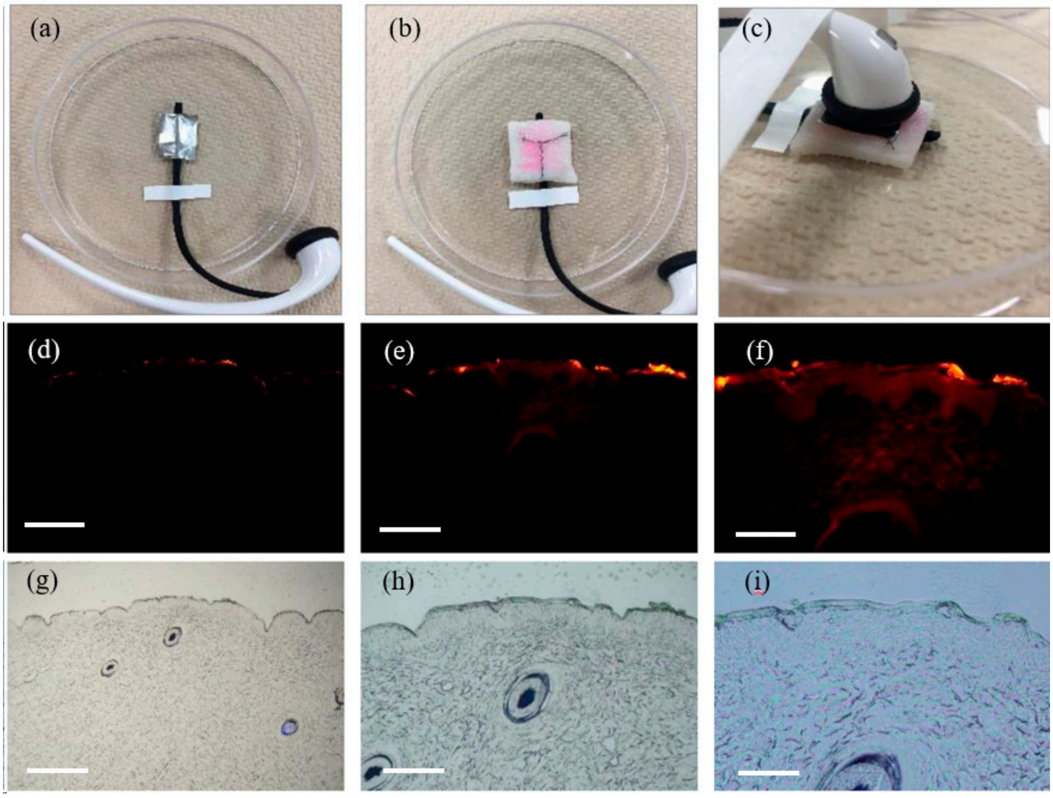

Figure S4. Electrode/FCM/Rhodamine B/electric mask pack composing device to observe the application effect of electric mask pack. (a)-(c) the device configuration steps of each experiment. (e,h) Control. (f,i) Needle scratch condition. (g,j) Electrically induced iontophoresis for transdermal transmission (Scale bars: 500 μm).

FCM showed low fluorescence by lowering the concentration of Rhodamine B. It was confirmed that high diffusion was observed in iontophoresis even when an electric mask pack was applied.

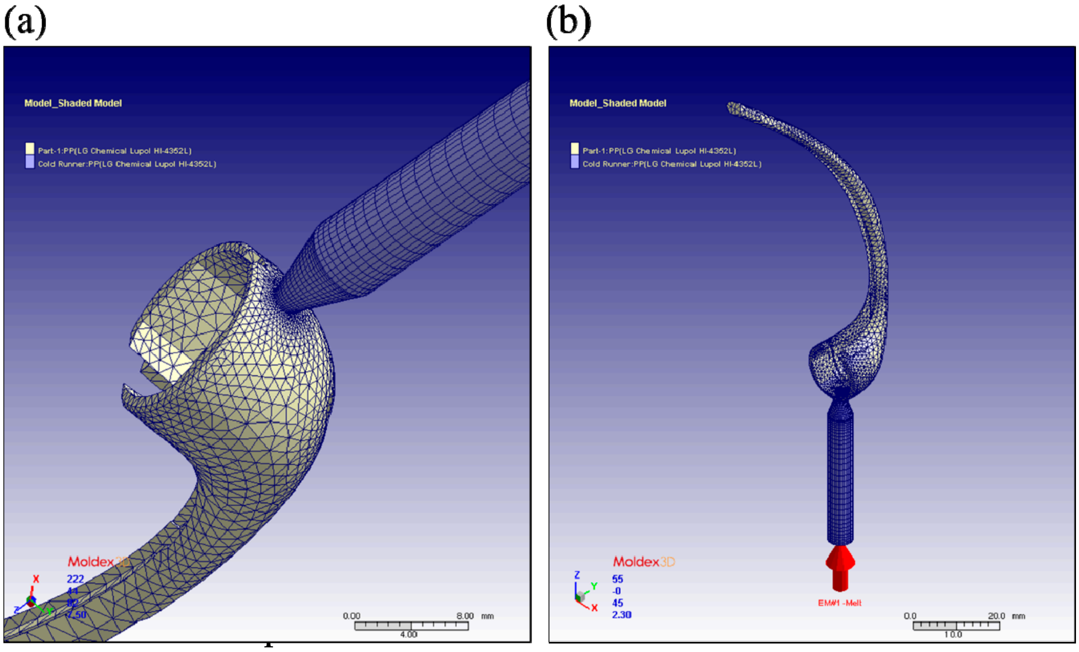

Figure S5. Mesh setting for the numerical analysis. (a) Gate part. (b) Entire mesh.

Figure S5 shows the mesh application part of the injection molding simulation. Figure S5(a) represents the gate part, whereas figure S5(b) is the entire mesh part.
